# The Application of TiO_2_/ZrO_2_-Modified Nanocomposite PES Membrane for Improved Permeability of Textile Dye in Water

**DOI:** 10.3390/membranes14100222

**Published:** 2024-10-21

**Authors:** Sibukiso Thobani Nhlengethwa, Charmaine Sesethu Tshangana, Bhekie Brilliance Mamba, Adolph Anga Muleja

**Affiliations:** Institute for Nanotechnology and Water Sustainability, College of Science, Engineering and Technology, University of South Africa, Johannesburg 1709, South Africa

**Keywords:** membrane rejection, nanoparticles, hydrophilicity, contaminated water, adsorption, photocatalysis

## Abstract

This study investigates the modification of polyethersulfone (PES) membranes with 1 wt% titanium dioxide (TiO_2_), zirconium dioxide (ZrO_2_) and a nanocomposite of TiO_2_/ZrO_2_. The aim was to efficiently remove Rhodamine B (RhB) from water using a threefold approach of adsorption, filtration and photodegradation. Among the modified membranes (TiO_2_, ZrO_2_ and TiO_2_/ZrO_2_), the TiO_2_/ZrO_2_-PES nanocomposite membrane showed a better performance in rejection of RhB than other membranes with the rejection efficiency of 96.5%. The TiO_2_/ZrO_2_-PES membrane was found to possess a thicker selective layer and reduced mean pore radius, which contributed to its improved rejection. The TiO_2_/ZrO_2_ nanocomposite membrane also showed high bulk porosity and a slightly lower contact angle of 69.88° compared to pristine PES with a value of 73°, indicating an improvement in hydrophilicity. Additionally, the TiO_2_/ZrO_2_-PES nanocomposite membrane demonstrated a relatively lower surface roughness (Sa) of 8.53 nm, which offers the membrane antifouling properties. The TiO_2_/ZrO_2_-PES membrane showed flux recovery ratio (FRR), total fouling (R_t_), reversible fouling (R_r_) and irreversible fouling (R_ir_) of 48.0%, 88.7%, 36,8% and 52.9%, respectively. For the photocatalytic degradation performance, the removal efficiency of RhB followed this order TiO_2_ > TiO_2_/ZrO_2_ > ZrO_2_ (87.6%, 85.7%, 67.8%). The tensile strength and elongation were found to be compromised with the addition of nanoparticles and nanocomposites. This indicates the necessity to further modify and optimise membrane fabrication to achieve improved mechanical strength of the membranes. At low pressure, the overall findings suggest that the TiO_2_/ZrO_2_ nanocomposite has the potential to offer significant improvements in membrane performance (water flux) compared to other modifications.

## 1. Introduction

Textile industries remain a significant cause of water pollution worldwide. The value of the textile dyes market globally reached USD 10.13 billion (in 2023) and is projected to grow to USD 17.23 billion by the year 2032 [1]. The major concern with the textile industry is the overconsumption of water and its environmental impact. According to the United Nations, the textile industry utilises an estimated 93 billion cubic metres of water and contributes approximately 20% of global wastewater annually [2]. Their effluent is comprised of microplastics, heavy metals and dyes. The textile industry releases 54% of dyed effluents in the environment amounting to 280,000 tons of textile dyes into water resources worldwide annually [3]. Textile effluent is often comprised of different classes of dyes including azo, anthraquinone, sulphur, phthalocyanine, indigo and xanthene dyes [4]. Rhodamine B (RhB), a highly stable synthetic organic dye belonging to the class of xanthene dyes known for imparting long-lasting bright pink colour in fabrics, has been used widely [5]. Due to the wide utilization of RhB in various industries, this dye is prone to unsafe disposal and release into the environment. Prolonged exposure to RhB has been associated with adverse effects on aquatic life, including fish and algae, the disruption of ecosystems and risk to biodiversity [6,7]. In humans, RhB is found to be carcinogenic, and mutagenic and can cause skin and eye irritation as well as respiratory problems [8,9]. Moreover, RhB is a prohibited colourant for usage in various consumer products due to its demonstrated carcinogenic, mutagenic and toxic properties, which warrants the development of efficient analytical methods [10] and removal technologies.

Currently, conventional water treatment systems in use have a considerable challenge in adequately removing dyes. Some treatment methods used to remove dyes from wastewater include advanced oxidation processes (AOPs), coagulation, adsorption, membranes and biological methods. However, most of these treatment methods are hindered either by long treatment periods, the generation of toxic by-products, large amounts of sludge produced or the need to regenerate spent adsorbents [11,12]. Membrane technology is one of the preferred technologies for treating dye-contaminated water. Polymeric membranes have exhibited good removal of dyes from wastewater. Polyether sulfone (PES)-based nanocomposite membranes are the most investigated in water treatment applications due to their excellent chemical and thermal stability, mechanical strength and the high permeability of PES [13,14,15].

Further, nanoparticles and nanocomposites have been successfully used for the treatment of dyes [16,17,18,19]. The addition of nanoparticles in polymeric membranes has been shown to enhance the rejection of dyes and improve membrane functions such as permeation and antifouling [20,21]. Several nanoparticles including titanium dioxide [22], zirconium dioxide [23], cobalt iron oxide [24], zinc oxide [25] and silicon dioxide [26] have been blended into membranes for different applications [27,28]. In the case where photocatalytic nanoparticles are incorporated, this imparts photocatalytic features to the membrane [29]. The strategy to incorporate various nanoparticles in the membranes is based on the anticipated positive synergistic effects and improved photocatalytic activity, as well as enhanced overall performance in the removal of dyes [15,30,31,32]. However, the adequate loading of the nanoparticles is a very crucial aspect to take into consideration in the design of these multi-functional membranes as a high nanoparticle loading may result in the blockage of pores and the agglomeration of nanoparticles within the membrane surfaces [13,21,28,29]. As such in this study, a 1 wt% titania–zirconia nanocomposite loading into a membrane will be investigated for dye removal from contaminated water. Moreover, this study explores the synergistic effects of titanium dioxide/zirconium dioxide (TiO_2_/ZrO_2_) nanocomposites in enhancing the properties and performance of the membranes by incorporating TiO_2_ nanoparticles, ZrO_2_ nanoparticles and TiO_2_/ZrO_2_ nanocomposites in PES membranes via mixed-matrix phase inversion methods to enhance membrane features such as permeability, rejection and photocatalytic degradation of RhB.

## 2. Experimental Methodology

### 2.1. Chemicals and Materials

All chemicals in this study were used as received commercially without any further purification. These include ethanol, titanium dioxide nanoparticles (TiO_2_), Zirconium (IV) oxychloride octahydrate (ZrOCl·8H_2_O), sodium hydroxide (NaOH), N-Methyl-2-pyrrolidone (NMP), Rhodamine B (RhB) and Bovine serum albumin (BSA), which were purchased from Merck (South Africa). While polyethersulfone (MW = 58,000 g/mol) was obtained from Solvay Polymers and Chemicals, South Africa. The Elix integral-10 water purification system from Merck Millipore was used in this study.

### 2.2. Synthesis Methods

#### 2.2.1. Synthesis of Titanium Dioxide (TiO_2_), Zirconium Dioxide (ZrO_2_) and TiO_2_/ZrO_2_ Nanocomposite

TiO_2_ nanoparticles were synthesized following a reported procedure [33], wherein 10 mL of titanium isopropoxide was added (dropwise) in 50 mL of deionized water to form hydrous titanium oxides (Ti(OH)_4_) under continuous stirring at 40 °C for 60 min resulting in a white precipitate. The white precipitate was separated through centrifugation and washed repeatedly with a mixture of deionized water and ethanol. The precipitate was dried at 80 °C in a drying oven (model DHG-9053A) for 12 h and subsequently calcined at 400 °C for 2 h at a ramping rate of 2.5 °C/min.

Zirconium dioxide nanoparticles were synthesised using an already-established method [34]. The ZrOCl.8H_2_O salt (4.00 g) was dissolved in 20 mL of deionized (DI) water and stirred until completely dissolved. A solution of 0.5 M NaOH was added drop wisely while stirring after which a white precipitate was formed. The precipitate was centrifuged and washed several times with DI water and ethanol. The product was dried overnight at 120 °C in the oven overnight and calcined at 450 °C for 1 h.

The TiO_2_/ZrO_2_ nanocomposites were fabricated using a previously reported physical mixing method [35]. For this study, a physical mixing method was selected and the interactions between nanoparticles are assumed to occur at the grain boundaries [35]. Appropriate masses of the TiO_2_ nanoparticles were physically mixed with ZrO_2_ nanoparticles at a ratio of 1:1. The mixed powder was thoroughly mixed using mortar and pestle. The finely ground mixture was subsequently used in the experiments without further treatment.

#### 2.2.2. Fabrication of PES Nanocomposite Membranes

The membranes were fabricated using the mixed-matrix phase inversion method [24]. The casting solutions were fabricated by dissolving PES (22 wt%), and 1 wt% nanoparticles (TiO_2_, ZrO_2_ and TiO_2_/ZrO_2_) in N-Methyl-2-pyrrolidone (77 wt%). The solutions were stirred with an overhead stirrer for 24 h until fully dissolved. The solutions were degassed by storing them in the dark for 24 h before being cast with the casting knife on the glass. The membranes were placed in a coagulation bath for 24 h after casting to ensure complete phase inversion. The fabricated membranes were named according to the nanoparticles loading: TiO_2_-PES, ZrO_2_-PES and TiO_2_/ZrO_2_-PES, and the control membrane in the study (without nanoparticles) is referred to as PES.

#### 2.2.3. Characterisation of the Prepared Materials

Nanomaterials were characterised using various techniques to obtain their structural, chemical and physical properties. An X-ray diffractometer (Rigaku SmartLab, Tokyo, Japan) with Cu-Kα (λ = 0.15406 nm) was employed to determine the crystalline structure and phase composition. Raman spectroscopy (Alpha 300A, WITec, GmbH, Ulm, Germany) was used to confirm the crystal phases of the nanoparticles. The functional groups of the nanoparticles were characterized with a Fourier-transform infrared (FTIR) spectrometer (Frontier PerkinElmer, spectrum 100 spectrometers, Waltham, MA, USA). Surface area and pore size distribution of nanoparticles were determined using Brunauer–Emmett–Teller (BET) N_2_ adsorption/desorption at a temperature of liquid nitrogen in a quantachrome autosorb IQ3 (autosorb IQ3, Boynton Beach, FL, USA) gas absorption analyser apparatus. Prior to the measurement, the samples were subjected to out-gassing at 100 °C for 1 h under a stream of N_2_. Scanning electron microscope and energy dispersive X-ray spectroscopy (SEM/EDS) instrument (JSM-7800F JEOL JEM-2100, Tokyo, Japan) assessed the morphological characteristics and elemental composition of the fabricated membranes. Atomic force microscopy (AFM) (Alpha300A, WITec, GmbH, Ulm, Germany) in non-contact mode was used to assess the membranes’ surface morphology and roughness (Sa). The hydrophilicity of the fabricated membranes was analysed by measuring the water contact angle (WCA) with a goniometer (DSA30 Krüss GmbH, Hamburg, Germany). The average WCA measurements were conducted using the static sessile drop method of 2 µL volume. UV–Vis Diffuse Reflectance Spectroscopy (Perkin Elmer UV–Vis spectrometer Lambda 6505, Changsha, Hunan, China) was employed to analyze the optical properties of TiO_2_, ZrO_2_ nanoparticles and TiO_2_/ZrO_2_ nanocomposites.

The bulk porosity and pore size of the fabricated membranes were estimated through the gravimetric method with the following Equations (1) and (2) [36].

(1) $$ℇ\left(\%\right)=\left(\frac{m_{wet}{-m}_{dry}}{A.l.\rho_{w}}\right)\times 100$$

where m_wet_ and m_dry_ represent the weight of the wet and dry membranes (g), respectively. A is the area of the membrane (cm^2^), l is the membrane’s thickness (cm) and ρ_w_ is the density of water at 0.998 g/cm^3^.

To measure porosity, the membranes (2 cm × 2 cm) were first soaked in distilled water for at least 24 h to ensure that all the pores in the membranes were filled with water. Afterwards, any water on the surface of the samples was slightly wiped and the membranes were weighed. Next, any water on the membranes was removed and then weighed again and the porosity was calculated.

The average pore radius of the membranes, r_m_, was estimated using the filtration method according to the Guerout–Elford–Ferry equation [37].

(2) $$r_{m}=\sqrt{\frac{\left(2.9-1.7ℇ\right)8Jl\eta }{ℇ.\Delta P}}$$

where J is water flux (m/s), η is the water viscosity (8.9 × 10^−4^ Pa·s) and ΔP is the operation pressure (4 bar = 400,000 Pa).

The mechanical properties of the membranes were identified using Small-Angle X-ray Scattering (SAXSpace) equipment (Anton Paar, Graz, Austria) with a tensile stage to measure strain and elongation.

### 2.3. Performance of Membranes

#### 2.3.1. Permeation of the Membranes

The water permeation of the membranes was evaluated by permeating Millipore water using a dead-end cell (Sterlitech, HP4750 stirred cell) with an effective membrane surface area of 0.00126 m^2^ and a volume of 250 mL. The water flux permeation was obtained by measuring the volume that a membrane permeates at a specific time at different pressures (4–6 bar); the membranes were not permeating from 0 to 3.5 bar. Before permeation, the membrane was pre-compacted for 15 min at 6 bars. To calculate the water flux, Equation (3) was applied [38].

(3) $$J_{W}=\frac{V}{A\Delta t}\;$$

where Jw is pure water flux (L/m^2^ h), V is the volume of water (L), A is the surface area of membrane (m^2^) and t is collection time (h).

#### 2.3.2. Rejection of Dyes

Rejection of RhB was assessed by quantifying the amount of dye that the membranes rejected from the feed solution at the operating pressure of 4 bar. The concentration of dye in the feed and permeate was determined by measuring the absorbance of RhB at the wavelength of maximum absorption (λ = 554 nm) with UV–Vis Spectroscopy (Perkin Elmer UV–Vis spectrometer Lambda 6505). The dye removal efficiency (R%) was determined using the Equation (4) [39].

(4) $$R\left(\%\right)=\left(1-\frac{C_{p}}{C_{f}}\right)\times 100$$

where C_f_ and C_p_ represent the concentration of the feed and the concentration of the permeate, respectively.

#### 2.3.3. Antifouling Behaviour of the Membranes

The solution of BSA (500 ppm; pH~7) was prepared and utilized as the fouling agent. The antifouling ability of the membranes was investigated by first doing the pure water flux (J_w1_) for 20 min, followed by the flux of BSA (J_BSA_) solution which was measured for 30 min with a sample collected every 15 min. Thereafter, the membrane was backwashed with deionized water for 30 min. After backwashing, pure water flux was repeated (J_w2_) for 20 min. To obtain fouling-resistant data, the flux recovery ratio (FRR) was calculated by applying Equation (5) [40].

(5) $$FRR(\%)=\frac{J_{w2}}{J_{w1}}\times 100$$

To analyse the fouling in detail, the total fouling (R_t_), reversible fouling (R_r_) and irreversible fouling (R_ir_) were evaluated by employing the following Equations (6)–(8), respectively [40].

(6) $$R_{t}\left(\%\right)=\left(1-\frac{J_{BSA}}{J_{w1}}\right)\times 100$$

(7) $$R_{r}\left(\%\right)=\left(\frac{{J_{w2}-J}_{BSA}}{J_{w1}}\right)\times 100$$

(8) $$R_{ir}(\%)=R_{t}\left(\%\right)-R_{r}(\%)$$

#### 2.3.4. Photocatalytic Degradation of RhB with Nanoparticles and the Fabricated Membranes

The photocatalytic activity of nanoparticles (TiO_2_ and ZrO_2_) and nanocomposite (TiO_2_/ZrO_2_) was tested by degrading RhB under UV radiation. A 30 mg sample of the nanoparticles or nanocomposite was added to 100 mL of 5 ppm RhB dye solution in a photoreactor. For the membranes, each membrane (4 cm × 4 cm) was immersed in a 100 mL dye solution of 5 ppm RhB and placed in a photoreactor (Lelesil Innovation Systems). The photoreactor is made of a 500 mL reaction vessel, 250 W UV lamp, a 4 L cooling tank, and a magnetic stirrer. Prior to irradiation with a UV lamp, the solution was stirred in the dark for 30 min to establish adsorption–desorption equilibrium between nanoparticles or nanocomposite membrane and the dye. In the evaluation of the photocatalytic activity of the nanoparticles and nanocomposite membranes, 5 mL samples were collected at 30 min intervals over 180 min. The concentrations of the dyes were analysed with a UV–Vis spectrometer at the wavelength of maximum absorption peak (λ = 554 nm). The extent of degradation was assessed by calculating the degraded dye using Equation (9) [15].

(9) $$Degraded\left(\%\right)=\left(\frac{{C_{i}-C}_{t}}{C_{i}}\right)\times 100$$

where C_t_ is the concentration at time and C_i_ is the initial concentration.

## 3. Results and Discussion

### 3.1. Characterisation of TiO_2_ and ZrO_2_ Nanoparticles

**Figure 1:**
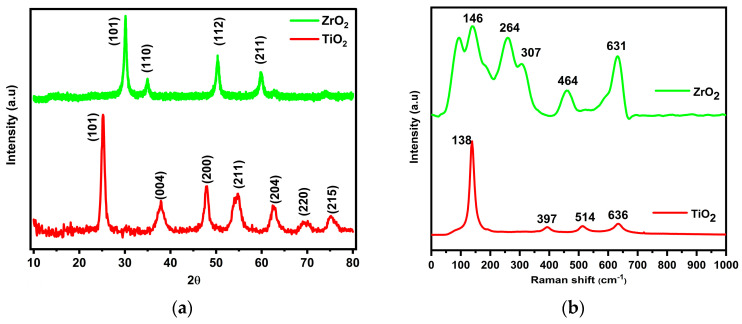

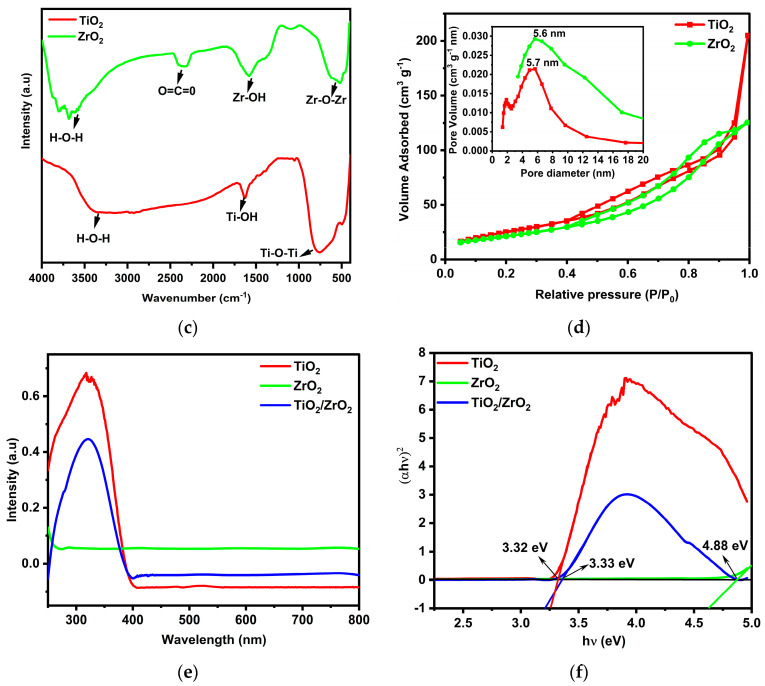
Various characterization of the synthesized TiO_2_ (red) and ZrO_2_ (green) nanoparticles: (**a**) XRD diffractogram, (**b**) Raman spectrum, (**c**) FTIR spectrum, (**d**) BET, (**e**) UV–vis DRS, and (**f**) Tauc plot of TiO_2_, ZrO_2_ nanoparticles and TiO_2_/ZrO_2_ nanocomposite.

The zirconia nanoparticles were characterized for various features and Figure 1a shows a diffractogram of the synthesized TiO_2_ and ZrO_2_ nanoparticles. The diffractogram of TiO_2_ nanoparticles (red) corresponds to the reported characteristic Miller indices in the crystal planes of the anatase phase of TiO_2_ (JCPDS-21-1272) [41]. The distinct peaks in the diffractogram of ZrO_2_ nanoparticles (green) correspond to the Miller indexes which defined the crystal planes of the tetragonal of ZrO_2_ (t-ZrO_2_) (JCPDS-89–7710) [34]. Raman spectra, as shown in Figure 1b, were used to further confirm the phase and crystal structure of TiO_2_ and ZrO_2_ nanoparticles. The spectrum of TiO_2_ (red) shows four characteristic peaks at 138 cm^−1^, 397 cm^−1^, 514 cm^−1^ and 636 cm^−1^. The peaks at 514 cm^−1^ and 636 cm^−1^ were assigned to the Ti-O stretching modes, the peak at 397 cm^−1^ was ascribed to O-Ti-O bending modes and 138 cm^−1^ was assigned to the primary peak of the anatase phase [42,43]. While ZrO_2_ nanoparticles (green) show the five bands at 146 cm^−1^, 264 cm^−1^, 307 cm^−1^, 464 cm^−1^ and 631 cm^−1^, confirming the structure of tetragonal zirconia [44]. The FTIR shown in Figure 1c shows broad absorption bands between 472 cm^−1^ and 900 cm^−1^ in the spectrum of TiO_2_ (red), and this was ascribed to the O-Ti-O bonding in the anatase phase of TiO_2_ [45]. While in the spectrum of ZrO_2_ (green), the band at 463 cm^−1^ depicts the Zr-O-Zr vibrations in the tetragonal structure of ZrO_2_. The observed bands at 3673 cm^−1^ and 1590 cm^−1^ are attributed to the O-H stretching and bending vibrations of the water adsorbed by both materials [34]. ZrO_2_ nanoparticles exhibited adsorption of atmospheric carbon dioxide with an absorption band at around 2353 cm^−1^. The surface area of TiO_2_ and ZrO_2_ were examined with desorption and adsorption isotherms, as shown in Figure 1d. Both isotherms resemble type-IV with the H_2_-type hysteresis loop, which indicates that both TiO_2_ and ZrO_2_ nanoparticles have a uniform size and mesoporous structure. The surface area was found to be 128.032 m^2^/g and 81.11 m^2^/g, respectively. The inset of Figure 1d shows a pore size distribution of TiO_2_ and t-ZrO_2_ nanoparticles, confirming the mesoporous structure with a pore diameter of 5.7 and 5.6 nm, respectively [46,47].

Figure 1e shows the DRS of TiO_2_ and ZrO_2_ nanoparticles and TiO_2_/ZrO_2_ nanocomposites. The DRS results indicate that TiO_2_ exhibited absorption in the ultraviolet range (λ < 400 nm), while ZrO_2_ demonstrated absorption in the deep ultraviolet region (λ < 300 nm). The TiO_2_/ZrO_2_ nanocomposite displayed absorption characteristics similar to that of pure TiO_2_. This suggests that the incorporation of ZrO_2_ did not alter the optical properties of TiO_2_ within the composite. This was also confirmed by the estimated band gap presented in the Tauc plot in Figure 1f, where TiO_2_ nanoparticles exhibited a band gap of 3.32 eV, closely aligning with the established value of approximately 3.2 eV for the anatase phase of TiO_2_ [48]. The band gap of the TiO_2_/ZrO_2_ nanocomposite was determined to be 3.33 eV, consistent with previously reported values of 3.36 eV for similar ZrO_2_-TiO_2_ photocatalysts [49]. While, ZrO_2_ nanoparticles demonstrated a band gap of 4.88 eV, which was comparable to the previously reported value of 4.99 eV [50]. These findings indicate that the introduction of ZrO_2_ into the TiO_2_ matrix minimally affects the optical properties or band gap of the composite, thereby preserving similar characteristics to pure TiO_2_ nanoparticles.

### 3.2. Membrane Characterisation

#### 3.2.1. Elemental Composition and Morphology

**Figure 2:**
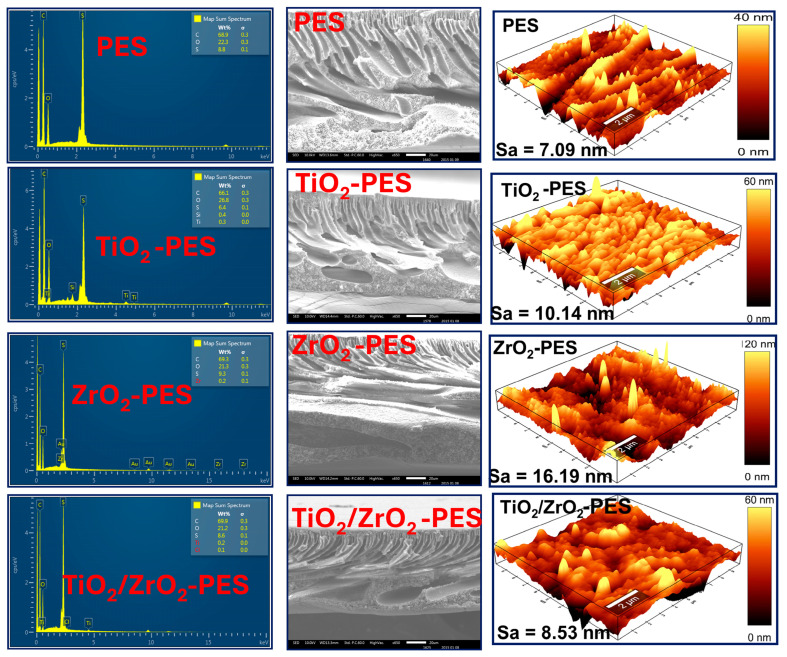
EDS spectra, SEM cross-sectional micrographs and AFM topographical micrographs of the pristine PES, TiO_2_-PES, ZrO_2_-PES, and TiO_2_/ZrO_2_-PES-modified membranes.

Figure 2 depicts the results of EDS analysis, SEM cross-sectional micrographs and AFM topographical micrographs of the membranes. The elemental compositions of the PES and nanocomposite membranes all contain the elements C, O and S, which correspond to carbon, oxygen and sulphur from the PES structure, respectively. In the membranes modified with 1 wt% TiO_2_, ZrO_2_ and TiO_2_/ZrO_2_, the metal percentage (Ti, and Zr) was found to be very low to be accurately quantified. They may be due to the uneven distribution of nanoparticles that occurs during the phase inversion process. During phase inversion, an uneven distribution of metal oxide nanoparticles within the polymer matrix can occur. As a result, on some regions of the membrane, certain elements may not be picked up or may be present but occur in low concentrations, making it difficult to detect during analysis. Consequently, if the sample is taken from an area with low element concentration, the EDS may fail to detect the element, leading to a reading indicating that the concentration is below the detection limit.

The SEM cross-sectional micrographs show that all the fabricated membranes exhibited the typical features of an asymmetric porous structure, consisting of a dense top layer and a porous sub-layer with a finger-like structure [51]. The modified membranes showed a decrease in the length of the finger-like structure but an increase in the thickness of the top later. This structural change was attributed to delayed de-mixing during the phase inversion process. Delayed de-mixing can occur when the addition of metal oxide nanoparticles alters the kinetics of solvent and non-solvent exchange. This alteration can slow down the formation of finger-like voids and promote the growth of a denser top layer [52]. The metal oxides also influence the polymer matrix, leading to these distinct morphological changes. The presence of metal oxides affects how the solvent diffuses out and the non-solvent diffuses into the polymer solution, resulting in a thicker, more compact top layer and shorter finger-like structures [53]. Among the modified membranes, modified TiO_2_/ZrO_2_ showed a relatively dense top layer. This signifies that the presence of TiO_2_/ZrO_2_ delayed the onset of water–NMP de-mixing, resulting in a denser top layer. This favours the application of the membrane in rejection, and membranes with a thicker selective layer turn out to be effective in rejection.

The surface roughness of the membranes is an important property that affects the membrane fouling behaviour by trapping fouling agents on the valleys (roughness) [54]. Figure 2 shows three-dimensional AFM images of the fabricated membranes with an average roughness (Sa). The pure PES membrane had a Sa of 7.09 nm. The incorporation of TiO_2_ increased the surface roughness to 10.14 nm, which was due to the agglomeration of nanoparticles. The membrane that was modified with ZrO_2_ had a higher surface roughness with Sa of 16.19 nm. However, the TiO_2_/ZrO_2_ composite resulted in membranes with less surface roughness with Sa of 8.53 nm. This indicates that the combination of ZrO_2_ and TiO_2_ resulted in a membrane better dispersion in a polymer matrix and a slower de-mixing process during the phase inversion of the TiO_2_/ZrO_2_ membrane, which results in a smoother membrane surface. Rapid de-mixing typically leads to the formation of more porous, finger-like structures and a rougher surface. In contrast, slower de-mixing promotes a more gradual and uniform phase separation, which can result in a denser and smoother membrane surface.

#### 3.2.2. Hydrophilicity, Bulk Porosity and Mean Pore Radius of the Fabricated Membranes

**Figure 3:**
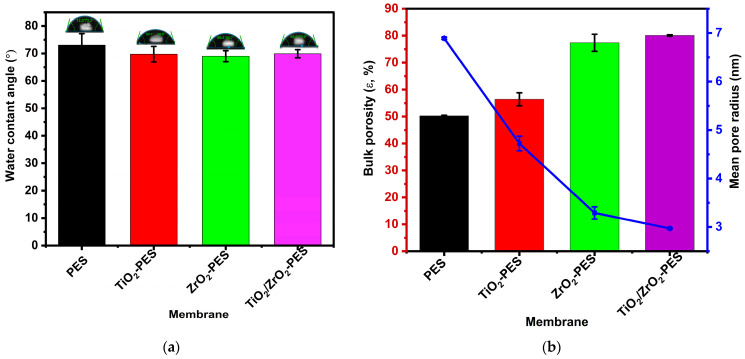
(**a**) Water contact angle and (**b**) bulk porosity and mean pore radius of the pristine PES, TiO_2_-PES, ZrO_2_-PES and TiO_2_/ZrO_2_-PES modified membranes.

The contact angle measures the hydrophobicity or hydrophilicity of the membrane. A lower contact angle signifies a greater hydrophilicity of the membrane. Generally, membranes with higher surface hydrophilicity are better at attracting water molecules and preventing the adsorption of contaminants. This is beneficial for enhancing water flux and preventing fouling, and the rejection of hydrophobic contaminants. Figure 3a shows the water contact angle of the fabricated membranes. The pure PES membrane showed a water contact angle of 73°. The water contact angles of all modified membranes were found to be slightly lower than that of pure PES membranes, indicating improvement in the hydrophilicity of the membranes. The contact angle of TiO_2_, ZrO_2_ and TiO_2_/ZrO_2_-modified membranes was found to be 69.8°, 69.10° and 70°, respectively.

The bulk porosity and mean pore radius of the membranes were determined by the gravimetric method. The gravimetric method allows for the direct calculation of the bulk porosity and can be used in conjunction with water permeation to estimate the mean pore radius of membranes [37]. Figure 3b shows the bulk porosity (bar graph) and mean pore radius (line). PES membrane was found to have a porosity of 50.25%, and the modified membranes show an increase in bulk porosity compared to the pristine PES membrane. The porosity of TiO_2_, ZrO_2_ and TiO_2_/ZrO_2_ membranes was found to be 56.41%, 77.38% and 80.08%, respectively. The improvement in the porosity of the modified membranes compared to pure PES membranes is due to the presence of hydrophilic nanoparticles that retain more water in the internal pores of the membranes. These results are complements of the WCA, which indicates that the nanoparticles improve the hydrophilicity of the membrane. However, among the modified membranes, TiO_2_/ZrO_2_-modified membranes showed a significant improvement in porosity followed by the ZrO_2_-modified membrane. This indicates that TiO_2_/ZrO_2_ retained more water in the internal pores of the membranes. Although the membranes showed an increase in bulk porosity with the addition of the nanoparticles, the mean pore radius of the modified membranes showed a decrease compared to the pristine PES membrane. The mean pore radius was found to be 6.89 nm, 4.72 nm, 3.29 nm and 2.97 nm for the PES, of TiO_2_, ZrO_2_ and TiO_2_/ZrO_2_ membranes, respectively. One can deduce that the loading of nanoparticles blocks the pores of the membranes, resulting in a reduced pore radius. The obtained mean pore radius of the fabricated membranes shows that the membranes are in the ultrafiltration range [55].

#### 3.2.3. Mechanical Strength

**Figure 4:**
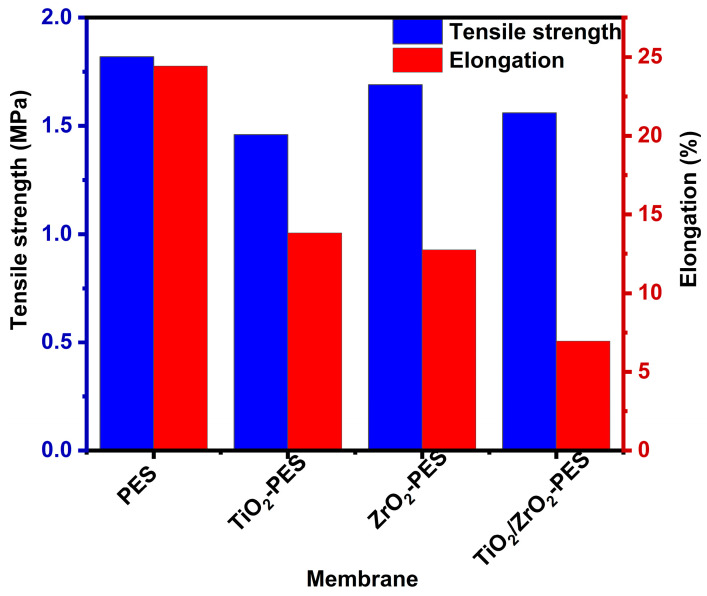
Tensile strength and elongation of the pristine PES, TiO_2_-PES, ZrO_2_-PES, and TiO_2_/ZrO_2_-PES membranes.

Mechanical strength tests including tensile strength and elongation were carried out and are shown in Figure 4. The tensile strength of the membrane is the maximum stress that a membrane can withstand while being stretched (elongated/strained) before breaking. The tensile strength of pure PES was found to be higher than that of the modified membrane, with values of 1.82, 1.46, 1.69 and 1.56 MPa for PES, TiO_2_, ZrO_2_ and TiO_2_/ZrO_2_-modified membranes, respectively. The reduction in tensile strength of membranes modified with nanoparticles can be attributed to the lack of strong chemical bonding between the inorganic nanoparticles and the organic polymer matrix. In pure PES, the polymer chains are free to align and distribute stress effectively when subjected to mechanical forces. This uniform stress distribution contributes to the higher tensile strength and greater elongation observed in pure PES. However, in the modified membranes, the inorganic nanoparticles are dispersed within the polymer but do not form significant covalent or other strong bonds with the polymer chains. This lack of interaction weakens the material’s ability to transfer and manage stress across its structure, resulting in lower tensile strength values for the modified membranes [56].

Similarly, with the elongation of the membranes, which measures the material’s ability to stretch before breaking, it was found to also decrease significantly when nanoparticles are introduced. In pure PES, TiO_2_, ZrO_2_ and TiO_2_/ZrO_2_-modified membranes were found to be 24.4%, 13.8%, 12.7% and 7.0%, respectively. This is because in pure PES, the polymer chain is free, and it allows the material to elongate more effectively under stress. However, the introduction of nanoparticles disrupts this chain flexibility. The rigid nanoparticles may also create physical defects that hinder the plasticity of the polymer chains, limiting the membrane’s ability to stretch and elongate [56]. Cazan et al. (2021) reported that the internal structure plays a crucial role in determining the mechanical properties of membranes. The addition of nanoparticles may cause agglomeration. These aggregates create defects in the nanocomposite, ultimately affecting its mechanical strength [57]. According to the results of tensile strength and elongation, the incorporation of nanoparticles into the PES matrix reduced the membranes’ mechanical properties due to the weak interaction between the polymer and the nanoparticles and defects that the nanoparticles may cause in the membranes.

### 3.3. Membranes Performances

#### 3.3.1. Water Flux, and Permeation of the Fabricated Membranes

**Figure 5:**
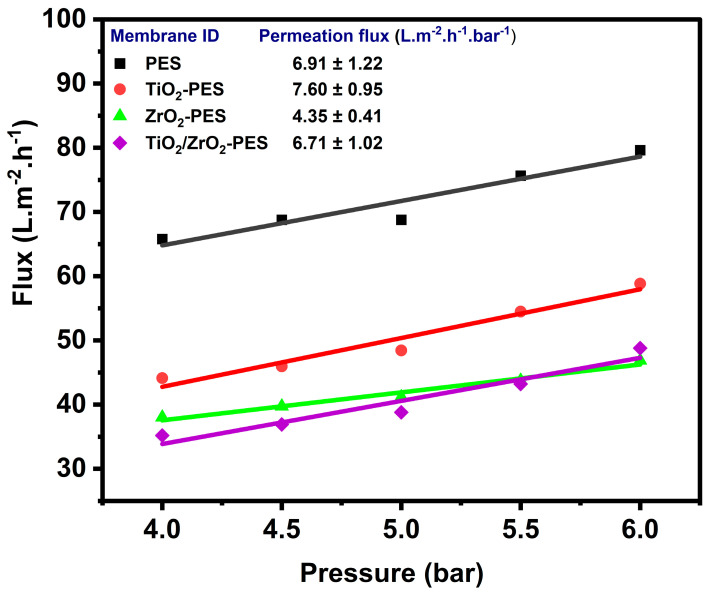
Water permeability of pristine PES, TiO_2_-PES, ZrO_2_-PES and TiO_2_/ZrO_2_-PES at different pressures, 5 min intervals while measuring the volume.

Water flux (J_w_) and permeability (L_P_) govern the rate at which water or other solvents pass through a membrane. Higher water flux and permeability lead to faster filtration rates, which are critical for efficient and cost-effective separation processes. These parameters are influenced by the membrane’s pore size, porosity, surface chemistry, thickness and structural properties. Water flux and permeability can be altered through the incorporation of nanoparticles in the membrane. Typically, the addition of hydrophilic nanoparticles into polymeric membranes improves the porosity, water flux and permeability of the membranes. However, from the obtained results of the plot of flux vs. pressure as shown in Figure 5, it was observed that the incorporation of the nanoparticles into the membrane matrix rather decreased the water flux of the membrane at different pressures. The observed reduction in water flux indicates that the incorporation of these nanoparticles significantly alters the membrane’s porosity and pore sizes. The introduction of TiO_2_, ZrO_2_ and TiO_2_/ZrO_2_ nanoparticles into the PES matrix resulted in reduced pore size, as seen previously in Figure 3b, which contributes to the significantly reduced water flux observed in the modified membranes. The water flux decreased in the following order: pure PES > TiO_2_ > ZrO_2_ >TiO_2_/ZrO_2_-modified membrane. The results obtained and the trend correspond to the pore radius reduction observed in Figure 3b.

However, the order of permeation, from highest to lowest was found to be TiO_2_-modified membranes, followed by pristine PES, TiO_2_/ZrO_2_-modified membranes, and finally ZrO_2_-modified membranes, with values of 7.60, 6.91, 6.71 and 4.35 L.m^−2^·h^−1^·bar^−1^, respectively. This suggests a varying impact of the different nanomaterials on the membrane structure. The TiO_2_-modified membranes had the highest permeability. While pristine PES and TiO_2_/ZrO_2_-modified membranes show a slight reduction in permeability, indicating that TiO_2_ nanoparticles may cause some reduction in pore size or lead to partial pore blocking. The combination of TiO_2_ and ZrO_2_ nanoparticles results in a slight reduction in permeability than TiO_2_ alone. ZrO_2_-modified membranes exhibit the most substantial decrease in permeability, likely due to the creation of the dense membrane structure resulting in a reduction in pore size.

These results suggest that the incorporation of nanoparticles leads to a denser membrane structure or smaller effective pore size, which reduces water fluxes and permeability. The ZrO_2_ nanoparticles in the membrane reduced the water flux and permeability far more significantly than the TiO_2_. The combination of TiO_2_ and ZrO_2_ provided an intermediate effect, indicating a balance between the impacts of the individual nanoparticles. This reduction in permeability could make these nanocomposite membranes more suitable for applications where selectivity and retention are more critical than high flux, such as in specific filtration or separation processes. This study emphasizes how the consideration and combination of selected nanoparticles can optimize membrane properties and form membranes with desired characteristics that are customized to specific applications.

#### 3.3.2. Rejection of RhB Dye

**Figure 6:**
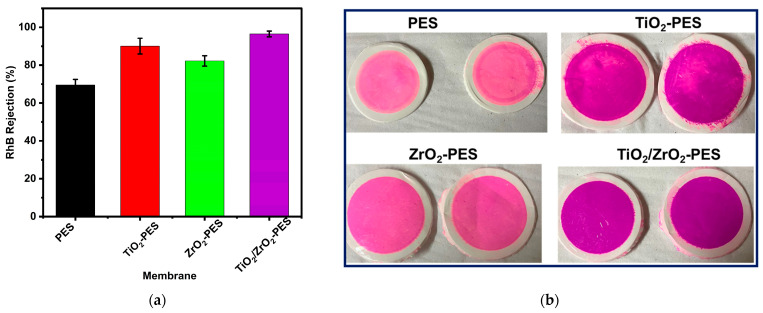
Rejection efficiency of RhB using pristine PES, TiO_2_-PES, ZrO_2_-PES and TiO_2_/ZrO_2_-PES membranes at 4 bar operating pressure.

In the rejection of RhB, as depicted in Figure 6a, TiO_2_ and ZrO_2_-modified membranes had higher rejection efficiency of RhB compared to that of pure PES (control). However, a more positive synergy obtained by adding the TiO_2_/ZrO_2_ nanocomposite resulted in an improved rejection ability of the membrane. Combining the two nanoparticles provided a synergetic effect to the membranes and enhanced the membranes’ rejection ability. The enhanced rejection TiO_2_/ZrO_2_-PES membrane could also be due to the dense selective layer observed in the SEM cross-section micrograph in Figure 2. The slightly lower rejection by the individual TiO_2_-PES and ZrO_2_-PES was attributed to the bigger pore size of the membranes observed in the gravimetric analysis as well as the nature and interaction of nanoparticles in the membranes and the RhB dye.

The superior rejection of RhB by TiO_2_/ZrO_2_-modified PES membranes was found to be 96.5%, followed by TiO_2_ and ZrO_2_-modified membranes with rejection rates of 90.1% and 82.1%, respectively, and finally pristine PES membranes with the rejection of 69.8%. This indicates that the specific combination of TiO_2_ and ZrO_2_ nanoparticles creates a more effective membrane for the rejection of RhB dye. The enhanced rejection observed in TiO_2_/ZrO_2_-modified membranes suggests that the synergistic effect of these nanoparticles results in a more favourable membrane structure for blocking RhB. This could be due to the formation of a denser, more uniform nanoparticle distribution within the PES matrix, leading to smaller effective pore sizes for dye molecules to pass through, thereby enhancing the membrane’s selectivity.

Figure 6b depicts pictures of used membranes after rejection of RhB, implying the varying rejection efficiencies (from light pink to deep purple colour). The pristine PES membrane appeared light pink after the test indicating less rejection, aligning with its rejection rate of 69.5%. In contrast, the TiO_2_-PES membrane displayed a deep purple colour signifying that most of the dye was successfully rejected, which is consistent with its high rejection efficiency of 90.1%. The ZrO_2_-PES membrane was a lighter purple indicating moderate rejection, corroborating the rejection efficiency of 82.3%. The TiO_2_/ZrO_2_-PES membrane exhibited a deep purple signifying a high degree of dye rejection matching its rejection rate of 96.5%. These pictures provide a clear indication of the membranes’ performance, correlating well with the quantitative rejection efficiency.

The results imply that while individual nanoparticle modifications may change the membrane’s permeability and rejection properties, it is the combination of TiO_2_ and ZrO_2_ that leads to a better RhB rejection. This combination likely results in a membrane with improved selectivity and rejection properties, making it more effective at filtering out dye molecules. The study indicates the importance of nanoparticle synergy in membrane design and suggests that combining different nanoparticles can lead to enhanced performance in specific applications, such as dye rejection in water treatment processes [58].

#### 3.3.3. Antifouling Behaviour of the Fabricated Membranes

**Figure 7:**
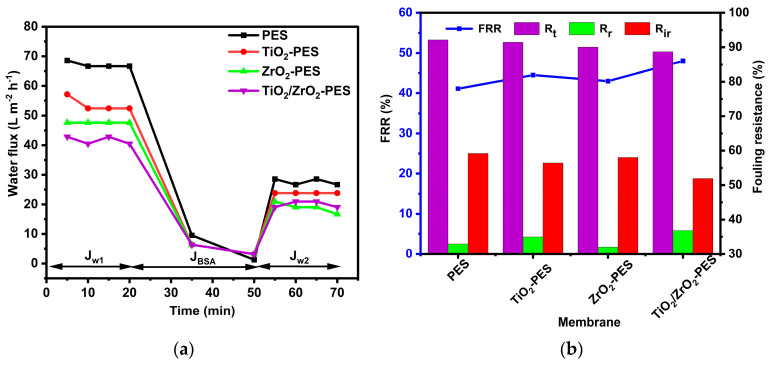
Antifouling behaviour of the pristine PES, TiO_2_, ZrO_2_ and TiO_2_/ZrO_2_-modified membranes (**a**) fouling with BSA and (**b**) flux recovery ratio (%) with fouling resistance ratios (%).

Figure 7a shows the antifouling behaviour of the pristine PES, TiO_2_-PES, ZrO_2_-PES and TiO_2_/ZrO_2_-PES membranes. From the water flux experiment, a sharp decline from over 40 L m^−2^ h^−1^ to below 10 L m^−2^ h^−1^ for all membranes suggested that BSA accumulated on the membrane surfaces (antifouling experiment), effectively blocking their pores and inhibiting water flow. However, when evaluating the flux recovery ratio (FRR), as shown in Figure 7b, the modified membranes demonstrated better flux recovery compared to the pristine PES membrane. The flux recovery ratios were 41.1%, 44.5%, 43% and 48.0% for the pristine PES, TiO_2_-PES, ZrO_2_-PES and TiO_2_/ZrO_2_-PES membranes, respectively. These results indicate that the incorporation of nanoparticles enhanced the antifouling performance of the membranes, particularly the TiO_2_/ZrO_2_ nanocomposite, which showed the highest recovery. The improved FRR suggests that the modified membranes had better antifouling properties.

The total fouling resistance (Rₜ) was found to be quite high with a value 92.1%, 91.4%, 90% and 88.7% for the pristine PES, TiO_2_-PES, ZrO_2_-PES and TiO_2_/ZrO_2_-PES membranes, respectively. The high values indicate that membranes were fouling during filtration of BSA. However, when the reversible fouling resistance (Rᵣ) was examined, it was found that a portion of the fouling is reversible in all membranes, indicating its ability to recover performance through physical cleaning. The reversible fouling resistance for the TiO_2_/ZrO_2_-PES membrane was found to be slightly higher compared to the other membranes, with a value of 36.8%. While pristine PES, TiO_2_-PES and ZrO_2_-PES were found to be 32.9%, 35.0% and 32.0%, respectively. In the irreversible fouling resistance (Rᵢᵣ), the TiO_2_/ZrO_2_-PES membrane showed the lowest Rᵢᵣ with a value of 51.9%, indicating less permanent fouling and a better ability to maintain long-term performance. In contrast, the PES and ZrO_2_-PES membranes exhibited comparatively higher irreversible fouling with a value of 59.2% and 58%, respectively. While TiO_2_-PES indicated moderate irreversible fouling with a value of 56.4%.

The enhanced resistance to fouling observed in the TiO_2_/ZrO_2_-PES membrane can be attributed to its improved surface smoothness and hydrophilicity. The smoother surfaces are highly advantageous for mitigating fouling as smoother surfaces are less likely to accumulate BSA [59]. Shang et al. (2020) reported that the membranes with a rougher surface are susceptive to fouling as the ridges and valleys on the surface of the membranes accumulate the foulants [60]. As illustrated in Figure 2, the surface roughness of the TiO_2_/ZrO_2_-modified membrane was found to be significantly smoother with a surface roughness of 8.53 nm, which was closer to that of a pristine PES membrane with 7.09 nm. However, the high fouling in the pristine membranes could be linked to the absence of nanoparticles, which makes them less hydrophilic. TiO_2_-PES had a moderated fouling resistance, which could be linked to its moderated surface roughness with a value of 10.14 nm. The less fouling resistance of ZrO_2_-PES could be linked to its higher surface roughness, which was found to be 16.18 nm. Hydrophilicity, another membrane feature, as indicated by the contact angle values in Figure 3a, further contributes to the membrane’s antifouling capabilities [52]. Sotto et al. (2014) reported that the significance of irreversible fouling as compared to reversible fouling between a pristine PES and TiO_2_-ZrO_2_-modified PES membrane was due to the presence of hydrophilic TiO_2_-ZrO_2_ nanocomposites [61].

#### 3.3.4. Photocatalytic Degradation of RhB with TiO_2_, ZrO_2_ and TiO_2_/ZrO_2_ Nanocomposite and Fabricated Membranes

**Figure 8:**
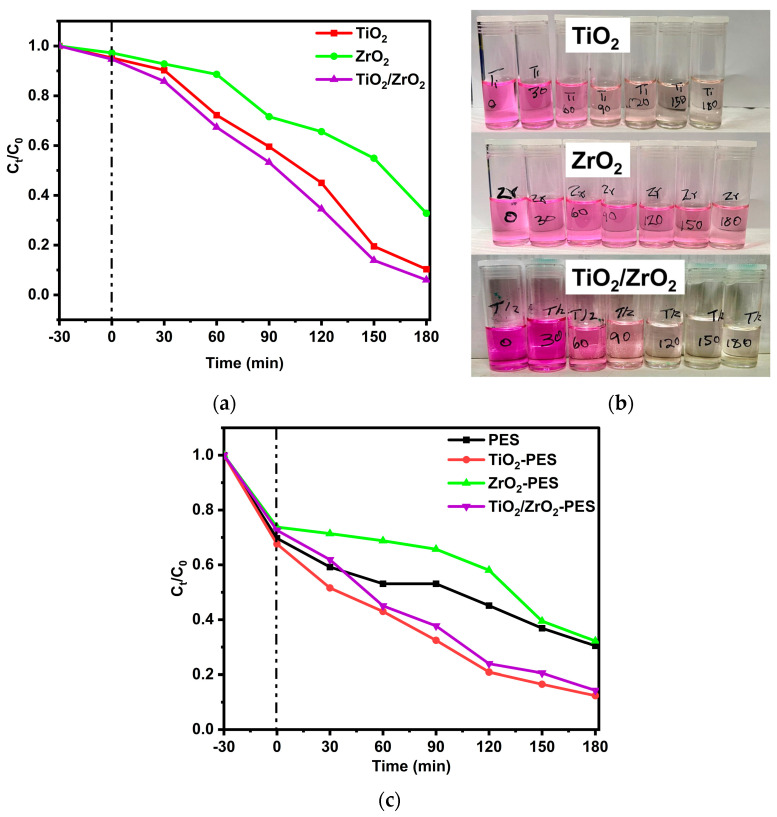
(**a**) Degradation curve of RhB with TiO_2_, ZrO_2_ and TiO_2_/ZrO_2_ nanocomposites; (**b**) degraded samples with TiO_2_, ZrO_2_ and TiO_2_/ZrO_2_ nanocomposites; and (**c**) degradation curve of RhB with pristine PES, TiO_2_-PES, ZrO_2_-PES and TiO_2_/ZrO_2_-PES-modified membranes under UV-light radiation for 3 h.

Figure 8a,b depict the degradation of RhB using TiO_2_, ZrO_2_ and TiO_2_/ZrO_2_ nanocomposites alongside the corresponding degraded dye water in sample vials. The degradation results show that TiO_2_ and TiO_2_/ZrO_2_ nanocomposites were highly effective in degrading RhB, achieving degradation rates of 89.7% and 96.0%, respectively. In comparison, ZrO_2_ nanoparticles demonstrated a low degradation efficiency of 67.2%. These findings suggest that the combination of TiO_2_ and ZrO_2_ significantly enhances photocatalytic activity, which could be attributed to the synergistic effect between the two nanoparticles. TiO_2_ is well-known for its photocatalytic properties under UV light, and the addition of ZrO_2_ appears to slightly improve the performance. In the observed UV–Vis DRS and band gap in the Tauc plot in Figure 1e,f, respectively, ZrO_2_ did not change the optical properties of TiO_2_ within the nanocomposites. Therefore, ZrO_2_ could improve its performance by accepting photogenerated electrons from TiO_2_ and exhibiting the electron-hole recombination [35]. The images shown in Figure 8b further reinforce these results.

The colour changes in the samples after degradation clearly show a substantial fading of RhB in the presence of TiO_2_ nanoparticles and TiO_2_/ZrO_2_ nanocomposites confirming their high photocatalytic efficiency of 89.7% and 96.0%, respectively. While the ZrO_2_ shows a less pronounced colour change, consistent with its lower degradation rate of 67.2%.

The photodegradation of RhB with the fabricated membranes is illustrated in Figure 8c. From these results, it can be observed that the TiO_2_/ZrO_2_-PES membranes performed similarly to TiO_2_-PES with a recorded RhB removal efficiency of 85.7% and 87.6%, respectively, while pristine PES and ZrO_2_-PES membranes also showed a comparable removal efficiency of 69.5% and 67.8%, respectively.

The significant decrease in the concentration of RhB indicates that the removal was more adsorption-controlled than photodegradation. The observation indicates adsorption as the dominant mechanism rather than photodegradation and suggests that the primary removal process of the dye is through surface adsorption onto the membranes. TiO_2_ and ZrO_2_ nanoparticles, known for their photocatalytic properties, also possess surfaces capable of adsorbing organic molecules like RhB [62]. In this case, it appears that the dye molecules undergo adsorption rather than undergoing breakdown through photocatalytic activity.

The chemical interactions between the RhB dye molecules and the membrane surfaces could be driving this adsorption process [63]. As a result, while the membranes are capable of adsorbing Rhodamine B effectively, the actual degradation of the dye through photocatalysis might be limited, pointing to adsorption as the more significant mechanism.

## 4. Conclusions

Understanding the effects of different nanoparticles and the loading of nanoparticles is crucial for the design and development of efficient membranes. This study demonstrated a positive synergy between TiO_2_/ZrO_2_ nanocomposites and PES in improving the permeability ability to remove RhB from water. Additionally, the TiO_2_/ZrO_2_-modified nanocomposite membrane demonstrated lower surface roughness, which offers the membrane antifouling properties. However, for the photocatalytic degradation performance, the efficiency followed this order TiO_2_ > ZrO_2_ > TiO_2_/ZrO_2_, implying that for the TiO_2_/ZrO_2_-modified nanocomposite membrane, the removal of RhB could be attributed to adsorption rather than photodegradation. The tensile strength and elongation were found to be compromised with the addition of nanoparticles and nanocomposites. This indicates the necessity to further modify and optimise membrane fabrication to achieve improved mechanical strength of the membranes. At low pressure, the overall findings suggest that the TiO_2_/ZrO_2_ nanocomposite has the potential to offer significant improvements in membrane performance (water flux) compared to other modifications. Future research should focus on addressing the challenges associated with nanoparticle (i) loading, (ii) surface distribution, and (iii) agglomeration in the modified membranes to enhance their photocatalytic performance. Optimizing the loading of nanoparticles is crucial, as higher or lower concentrations can affect both photocatalytic activity and mechanical properties. Varying the loading could help identify the concentration that maximizes surface availability on the membrane for photocatalysis. Alternative surface modification techniques such as UV-induced grafting should be explored to improve the uniform distribution of nanoparticles on the membrane surface. These methods will ensure greater exposure of nanoparticles to light, enhancing photocatalytic efficiency and making them suitable for the degradation of dyes.

## Data Availability

The original contributions presented in the study are included in the article, further inquiries can be directed to the corresponding author.

## References

[1] Global Textile Dyes Market to Grow at a CAGR of 6% in the Forecast Period of 2024–2032. https://www.expertmarketresearch.com/reports/textile-dyes-market.

[2] United Nations UN launches drive to highlight environmental cost of staying fashionable, UN News, 25 March 2019. https://news.un.org/en/story/2019/03/1035161.

[3] Jorge A.M.S., Athira K.K., Alves M.B., Gardas R.L., Pereira J.F.B. (2023). Textile dyes effluents: A current scenario and the use of aqueous biphasic systems for the recovery of dyes. J. Water Process Eng..

[4] Ahmad A., Jawaid M., Ibrahim M.N.M., Yaqoob A.A., Alshammari M.B. (2023). Nanohybrid Materials for Treatment of Textiles Dyes.

[5] Waghchaure R.H., Adole V.A., Jagdale B.S. (2022). Photocatalytic degradation of methylene blue, rhodamine B, methyl orange and Eriochrome black T dyes by modified ZnO nanocatalysts: A concise review. Inorg. Chem. Commun..

[6] Oladoye P.O., Kadhom M., Khan I., Hama Aziz K.H., Alli Y.A. (2023). Advancements in adsorption and photodegradation technologies for Rhodamine B dye wastewater treatment: Fundamentals, applications, and future directions. Green Chem. Eng..

[7] Sudarshan S., Bharti V.S., Harikrishnan S., Shukla S.P., RathiBhuvaneswari G. (2022). Eco-toxicological effect of a commercial dye Rhodamine B on freshwater microalgae *Chlorella vulgaris*. Arch. Microbiol..

[8] Saigl Z. (2021). Various Adsorbents for Removal of Rhodamine B Dye: A Review. Indones. J. Chem..

[9] Sundararajan M., Bonisha B., Ubaidullah M., Shaikh S.M.F., Revathi S., Thiripurasundari D., Dhiwahar A.T., Pandit B., Dash C.S., Shahazad M. (2022). Enhanced visible light photocatalytic degradation of rhodamine B using Ni_1−x_Ca_x_Fe_2_O_4_ (0 ≤ x ≤ 0.5) nanoparticles: Performance, kinetics and mechanism. Mater. Res. Bull..

[10] Du R.-Z., Zhang Y., Bian Y., Yang C.-Y., Feng X.-S., He Z.-W. (2024). Rhodamine and related substances in food: Recent updates on pretreatment and analysis methods. Food Chem..

[11] Abd-Elhamid A.I., Kamoun E.A., El-Shanshory A.A., Soliman H.M.A., Aly H.F. (2019). Evaluation of graphene oxide-activated carbon as effective composite adsorbent toward the removal of cationic dyes: Composite preparation, characterization and adsorption parameters. J. Mol. Liq..

[12] Streit A.F.M., Côrtes L.N., Druzian S.P., Godinho M., Collazzo G.C., Perondi D., Dotto G.L. (2019). Development of high quality activated carbon from biological sludge and its application for dyes removal from aqueous solutions. Sci. Total Environ..

[13] Otitoju T.A., Ahmad A.L., Ooi B.S. (2018). Recent advances in hydrophilic modification and performance of polyethersulfone (PES) membrane via additive blending. RSC Adv..

[14] Ashok Kumar S., Srinivasan G., Govindaradjane S. (2019). Development of a new blended polyethersulfone membrane for dye removal from synthetic wastewater. Environ. Nanotechnol. Monit. Manag..

[15] Muleja A.A., Mamba B.B. (2018). Development of calcined catalytic membrane for potential photodegradation of Congo red in aqueous solution. J. Environ. Chem. Eng..

[16] Muleja A.A., Mubiayi M.P., Hassard F., Mamba B.B. (2021). Titania containing natural clay doped with carbon nanotubes for enhanced natural photocatalytic discoloration of wastewater. J. Nanopart. Res..

[17] Tshangana C.S., Muleja A.A., Mamba B.B. (2022). Photocatalytic activity of graphene oxide quantum dots in an effluent from a South African wastewater treatment plant. J. Nanopart. Res..

[18] Sanchez L.M., Ollier R.P., Gonzalez J.S., Alvarez V.A., Mustansar Hussain C. (2018). Chapter 51—Nanocomposite Materials for Dyes Removal. Handbook of Nanomaterials for Industrial Applications.

[19] Gomes R.F., de Azevedo A.C.N., Pereira A.G.B., Muniz E.C., Fajardo A.R., Rodrigues F.H.A. (2015). Fast dye removal from water by starch-based nanocomposites. J. Colloid. Interface Sci..

[20] Ursino C., Castro-Muñoz R., Drioli E., Gzara L., Albeirutty M.H., Figoli A. (2018). Progress of Nanocomposite Membranes for Water Treatment. Membranes.

[21] Kusworo T.D., Nugraheni R.E., Aryanti N. (2021). The Effect of Membrane Modification Using TiO_2_, ZnO, and GO Nanoparticles: Challenges and Future Direction in Wastewater Treatment. IOP Conf. Ser. Mater. Sci. Eng..

[22] Arif Z., Sethy N.K., Kumari L., Mishra P.K., Verma B. (2019). Antifouling behaviour of PVDF/TiO_2_ composite membrane: A quantitative and qualitative assessment. Iran. Polym. J..

[23] Chen X., Huang G., An C., Feng R., Wu Y., Huang C. (2022). Superwetting polyethersulfone membrane functionalized with ZrO_2_ nanoparticles for polycyclic aromatic hydrocarbon removal. J. Mater. Sci. Technol..

[24] Chakachaka V.M., Mahlangu O.T., Tshangana C.S., Mamba B.B., Muleja A.A. (2023). Highly adhesive CoFe_2_O_4_ nanoengineered PES membranes for salts and Naproxen removal and antimicrobial activities. J. Membr. Sci..

[25] Shen L., Huang Z., Liu Y., Li R., Xu Y., Jakaj G., Lin H. (2020). Polymeric Membranes Incorporated with ZnO Nanoparticles for Membrane Fouling Mitigation: A Brief Review. Front. Chem..

[26] Muhamad M.S., Salim M.R., Lau W.-J. (2015). Surface modification of SiO_2_ nanoparticles and its impact on the properties of PES-based hollow fiber membrane. RSC Adv..

[27] Ng L.Y., Mohammad A.W., Leo C.P., Hilal N. (2013). Polymeric membranes incorporated with metal/metal oxide nanoparticles: A comprehensive review. Desalination.

[28] Iqbal A., Cevik E., Mustafa A., Qahtan T.F., Zeeshan M., Bozkurt A. (2024). Emerging developments in polymeric nanocomposite membrane-based filtration for water purification: A concise overview of toxic metal removal. Chem. Eng. J..

[29] Esfahani M.R., Aktij S.A., Dabaghian Z., Firouzjaei M.D., Rahimpour A., Eke J., Escobar I.C., Abolhassani M., Greenlee L.F., Esfahani A.R. (2019). Nanocomposite membranes for water separation and purification: Fabrication, modification, and applications. Sep. Purif. Technol..

[30] Nasrollahi N., Ghalamchi L., Vatanpour V., Khataee A. (2021). Photocatalytic-membrane technology: A critical review for membrane fouling mitigation. J. Ind. Eng. Chem..

[31] Abdullah R.R., Shabeed K.M., Alzubaydi A.B., Alsalhy Q.F. (2022). Novel photocatalytic polyether sulphone ultrafiltration (UF) membrane reinforced with oxygen-deficient Tungsten Oxide (WO2.89) for Congo red dye removal. Chem. Eng. Res. Des..

[32] Ding C., Qin X., Tian Y., Cheng B. (2022). PES membrane surface modification via layer-by-layer self-assembly of GO@TiO_2_ for improved photocatalytic performance. J. Membr. Sci..

[33] Buraso W., Lachom V., Siriya P., Laokul P. (2018). Synthesis of TiO_2_ nanoparticles via a simple precipitation method and photocatalytic performance. Mater. Res. Express.

[34] Anandan K., Rajesh K., Gayathri K., Vinoth Sharma S., Mohammed Hussain S.G., Rajendran V. (2020). Effects of rare earth, transition and post transition metal ions on structural and optical properties and photocatalytic activities of zirconia (ZrO_2_) nanoparticles synthesized via the facile precipitation process. Phys. E Low-Dimens. Syst. Nanostruct..

[35] Polisetti S., Deshpande P.A., Madras G. (2011). Photocatalytic Activity of Combustion Synthesized ZrO_2_ and ZrO_2_–TiO_2_ Mixed Oxides. Ind. Eng. Chem. Res..

[36] Li J.-F., Xu Z.-L., Yang H., Yu L.-Y., Liu M. (2009). Effect of TiO_2_ nanoparticles on the surface morphology and performance of microporous PES membrane. Appl. Surf. Sci..

[37] Díez B., Santiago-Morales J., Martínez-Bueno M.J., Fernández-Alba A.R., Rosal R. (2017). Antimicrobial organic–inorganic composite membranes including sepiolite-supported nanometals. RSC Adv..

[38] Mohamat R., Bakar S.A., Mohamed A., Muqoyyanah M., Othman M.H.D., Mamat M.H., Malek M.F., Ahmad M.K., Yulkifli Y., Ramakrishna S. (2023). Incorporation of graphene oxide/titanium dioxide with different polymer materials and its effects on methylene blue dye rejection and antifouling ability. Environ. Sci. Pollut. Res..

[39] Li X., Fang X., Pang R., Li J., Sun X., Shen J., Han W., Wang L. (2014). Self-assembly of TiO_2_ nanoparticles around the pores of PES ultrafiltration membrane for mitigating organic fouling. J. Membr. Sci..

[40] Ma D., Zou X., Zhao Z., Zhou J., Li S., Yin H., Wang J. (2022). Hydrophilic PAA-g-MWCNT/TiO_2_@PES nano-matrix composite membranes: Anti-fouling, antibacterial and photocatalytic. Eur. Polym. J..

[41] Einert M., Hartmann P., Smarsly B., Brezesinski T. (2021). Quasi-homogenous photocatalysis of quantum-sized Fe-doped TiO_2_ in optically transparent aqueous dispersions. Sci. Rep..

[42] Hardcastle F.D. (2011). Raman Spectroscopy of Titania (TiO_2_) Nanotubular Water-Splitting Catalysts. J. Ark. Acad. Sci..

[43] Kernazhitsky L., Shymanovska V., Gavrilko T., Naumov V., Fedorenko L., Kshnyakin V., Baran J. (2014). Laser-excited excitonic luminescence of nanocrystalline TiO_2_ powder. Ukr. J. Phys..

[44] Basahel S.N., Ali T.T., Mokhtar M., Narasimharao K. (2015). Influence of crystal structure of nanosized ZrO_2_ on photocatalytic degradation of methyl orange. Nanoscale Res. Lett..

[45] Al-Oubidy E.A., Kadhim F.J. (2019). Photocatalytic activity of anatase titanium dioxide nanostructures prepared by reactive magnetron sputtering technique. Opt. Quantum Electron..

[46] Fan Z., Meng F., Gong J., Li H., Ding Z., Ding B. (2016). One-step hydrothermal synthesis of mesoporous Ce-doped anatase TiO_2_ nanoparticles with enhanced photocatalytic activity. J. Mater. Sci. Mater. Electron..

[47] Rahman N.J.A., Ramli A., Jumbri K., Uemura Y. (2019). Tailoring the surface area and the acid–base properties of ZrO_2_ for biodiesel production from *Nannochloropsis* sp.. Sci. Rep..

[48] Ahmed T.Y., Abdullah O.G., Mamand S.M., Aziz S.B. (2024). Band structure study of pure and doped anatase titanium dioxide (TiO_2_) using first-principle-calculations: Role of atomic mass of transition metal elements (TME) on band gap reduction. Opt. Quantum Electron..

[49] Yaacob N., Ismail A.F., Sean G.P., Nazri N.A.M. (2019). Structural and photocatalytic properties of co-doped hybrid ZrO_2_–TiO_2_ photocatalysts. SN Appl. Sci..

[50] Wu H., Duan Y., Liu K., Lv D., Qin L., Shi L., Tang G. (2015). First-principles study of phase transition and band structure of ZrO_2_ under pressure. J. Alloys Compd..

[51] Pereira V.R., Isloor A.M., Ahmed A.A., Ismail A.F. (2015). Preparation, characterization and the effect of PANI coated TiO_2_ nanocomposites on the performance of polysulfone ultrafiltration membranes. New J. Chem..

[52] Mahlangu O.T., Nackaerts R., Thwala J.M., Mamba B.B., Verliefde A.R.D. (2017). Hydrophilic fouling-resistant GO-ZnO/PES membranes for wastewater reclamation. J. Membr. Sci..

[53] Hołda A.K., Vankelecom I.F.J. (2015). Understanding and guiding the phase inversion process for synthesis of solvent resistant nanofiltration membranes. J. Appl. Polym. Sci..

[54] Zhong Z., Li D., Zhang B., Xing W. (2012). Membrane surface roughness characterization and its influence on ultrafine particle adhesion. Sep. Purif. Technol..

[55] Yang Z., Zhou Y., Feng Z., Rui X., Zhang T., Zhang Z. (2019). A Review on Reverse Osmosis and Nanofiltration Membranes for Water Purification. Polymers.

[56] Alghamdi M.N. (2016). Titanium Dioxide Reinforced Polypropylene Composites: Preparation and Characterization. Int. J. Eng. Res. Technol..

[57] Cazan C., Enesca A., Andronic L. (2021). Synergic Effect of TiO_2_ Filler on the Mechanical Properties of Polymer Nanocomposites. Polymers.

[58] Yin Y., Wang H., Li D., Jing W., Fan Y., Xing W. (2016). Fabrication of mesoporous titania–zirconia composite membranes based on nanoparticles improved hydrosol. J. Colloid. Interface Sci..

[59] Hoek E.M.V., Bhattacharjee S., Elimelech M. (2003). Effect of Membrane Surface Roughness on Colloid−Membrane DLVO Interactions. Langmuir.

[60] Shang C., Pranantyo D., Zhang S. (2020). Understanding the Roughness–Fouling Relationship in Reverse Osmosis: Mechanism and Implications. Environ. Sci. Technol..

[61] Sotto A., Kim J., Arsuaga J., Del Rosario G., Martínez A., Nam D., Luis P., Van der Bruggen B. (2014). Binary metal oxides for composite ultrafiltration membranes. J. Mater. Chem. A.

[62] Das L., Basu J.K. (2015). Photocatalytic treatment of textile effluent using titania–zirconia nano composite catalyst. J. Ind. Eng. Chem..

[63] Xu Y., Lin W., Wang H., Guo J., Yuan D., Bao J., Sun S., Zhao W., Zhao C. (2020). Dual-functional polyethersulfone composite nanofibrous membranes with synergistic adsorption and photocatalytic degradation for organic dyes. Compos. Sci. Technol..

